# Awareness and willingness to use HIV self-testing among people who inject drugs in Iran

**DOI:** 10.1186/s12954-023-00881-z

**Published:** 2023-10-07

**Authors:** Mehrdad Khezri, Emily Goldmann, Fatemeh Tavakoli, Mohammad Karamouzian, Mostafa Shokoohi, Soheil Mehmandoost, Nima Ghalekhani, Ali Akbar Haghdoost, Don Des Jarlais, Ali Mirzazadeh, Hamid Sharifi

**Affiliations:** 1 Department of Epidemiology, New York University School of Global Public Health, New York, NY USA; 2 HIV/STI Surveillance Research Center, and WHO Collaborating Center for HIV Surveillance, Institute for Futures Studies in Health, Kerman University of Medical Sciences, Kerman, Iran; 3 Center for Drug Use and HIV/HCV Research, New York, NY USA; 4 Centre on Drug Policy Evaluation, MAP Centre for Urban Health Solutions, St. Michael’s Hospital, Toronto, ON Canada; 5 Dalla Lana School of Public Health, University of Toronto, Toronto, ON Canada; 6 Department of Health Sciences, Faculty of Applied Health Sciences, Brock University, St. Catharines, ON Canada; 7 Department of Epidemiology and Biostatistics, University of California, San Francisco, CA USA

**Keywords:** HIV, HIV self-testing, People who inject drugs, Harm reduction, Iran

## Abstract

**Background:**

Most people who inject drugs (PWID) in Iran have not undergone recent HIV testing. While PWID face barriers when seeking HIV testing at health facilities, HIV self-testing (HIVST) could be a promising approach to improve HIV testing uptake. We examined the awareness and willingness to use HIVST among PWID in Iran. We also identified participants’ characteristics associated with a higher willingness to use HIVST.

**Methods:**

PWID were recruited in 11 cities using a respondent-driven sampling method. Willingness to use HIVST was defined as a binary variable (very low/low willingness vs. high/very high willingness). We performed multivariable modified Poisson regression to examine associated factors and report adjusted prevalence ratios (aPR) and 95% confidence intervals (CI).

**Results:**

Of 2,252 PWID, 362 (16.2%; 95% CI 14.7, 17.8) had ever heard of HIVST; however, 1,658 (73.6%; 95% CI 71.7, 75.4) reported high/very high willingness to use HIVST. Willingness to use HIVST was higher among PWID who reported having a high/moderate HIV risk perception (aPR 1.22; 95% CI 1.09, 1.37), ever experiencing homelessness (aPR 1.15; 95% CI 1.03, 1.28), > 10 years of injecting history (aPR 1.16; 95% CI 1.00, 1.34), and high injection frequency in the last three months (aPR 1.18; 95% CI 1.05, 1.32).

**Conclusion:**

Most PWID in Iran, particularly those experiencing homelessness, have a longer injecting history, engage in more frequent injection practices, and possess a heightened perception of HIV risk would be willing to adopt HIVST. Enhancing HIVST awareness through increased access to HIVST and health education programs are needed. Additionally, conducting implementation science studies to effectively design and run HIVST programs in Iran can also increase PWID’s access to HIV testing.

**Supplementary Information:**

The online version contains supplementary material available at 10.1186/s12954-023-00881-z.

## Introduction

Iran has an estimated 345,308 people who inject drugs (PWID) [1], most of whom are at risk of HIV due to unmet treatment and prevention needs, including a low level of HIV testing [2]. For example, a study in the capital city of Tehran reported that over one-third of PWID had never tested for HIV in 2018 [3]. Another study showed that among PWID who tested positive for HIV, one-third were unaware of their HIV status [4]. A national study in 2010 also indicated that over half of PWID in Iran had never tested for HIV, and only one-fourth had tested for HIV in the previous year [2]. The low level of HIV testing in Iran has been also reported among other populations at risk for HIV, including female sex workers [5] and incarcerated people [6]. In addition, a recent study using data from the HIV Case Registry System of Iran estimated that among the 59,314 people living with HIV in Iran, only 37% (22,054) were diagnosed, indicating testing and diagnosis as the primary gap in the HIV cascade of care in Iran [7].

HIV self-testing (HIVST) is a diagnostic test where individuals collect their own specimens and perform the test themselves [8]. Despite concerns about the absence of pre-and post-test counseling, confirmatory testing, linkage to further HIV testing and diagnosis, and the window period (i.e., the time between HIV exposure and when a test can identify HIV) [9], several studies have demonstrated that HIVST has promising results and could facilitate  increased HIV testing uptake worldwide [10]. This may be due to the high level of acceptability of HIVST among individuals who might not otherwise test for HIV, including those who worry about the stigma associated with undergoing an HIV test at a healthcare facility and those who have difficulty finding the time to go to a clinic for testing [11]. Studies suggest that HIVST could offer healthcare systems a more practical means to provide greater accessibility, lower costs, and better adherence [12, 13]. Furthermore, HIVST offers a pathway for marginalized populations or individuals with limited healthcare system access, such as sexual and gender minority communities, rural populations, and PWID [8].

Despite the numerous benefits associated with HIVST, it has not yet been integrated into Iran’s HIV prevention and treatment programs. HIV prevention and harm reduction programs for PWID in Iran usually consist of services, such as opioid agonist therapy, distribution of sterile needles and syringes at no cost, provision of free condoms, and educational programs that cover sexual health and safe injection practices [14, 15]. Free rapid HIV testing, counseling, and referral are also available through drop-in centers and voluntary counseling and testing centers [15]. Additionally, research on the implementation and evaluation of HIVST in Iran also remains limited to studies with small sample sizes and among other key populations, including men who have sex with men, transgender people, and female sex workers [16, 17]. In response, we aimed to examine the level of awareness and willingness to use HIVST among PWID in Iran, with the estimated HIV prevalence ranged from 3.5% to 15.1% during the last decade [18]. We also identified participants’ characteristics associated with a higher willingness to use HIVST among a large and nationally representative sample of PWID in Iran.

## Methods

### Study design, setting, and participants

This study used data collected as part of the fourth national HIV bio-behavioral surveillance survey among PWID in Iran. Details of this survey are described elsewhere [18–20]. In brief, we recruited PWID in 11 major cities in Iran, including Tehran (central north), Tabriz (northwest), Sari (north), Mashhad (northeast), Yazd (central), Kermanshah (west), Khorramabad (west), Ahvaz (southwest), Shiraz (south), Kerman (southeast) and Zahedan (southeast). We selected cities following consultations with Iran’s Ministry of Health and Medical Education to represent different regions across the country. Inclusion criteria for enrollment were: (1) being at least 18 years old, (2) having self-reported injection of any drugs at least once in the past 12 months, and (3) having a valid referral coupon consistent with the study methodology.

### Sampling and data collection

We recruited participants between July 2019 and March 2020 using respondent-driven sampling (RDS), a chain-referral recruitment approach used in HIV surveillance and research to identify and enroll marginalized populations, including PWID [21, 22]. Initial recruits (i.e., seeds) from various subgroups of PWID with expansive social networks were recruited to initiate recruitment. We provided three coupons to each seed to distribute to social network peers with an expiration date of three weeks. Participants received monetary compensation for the interview and HIV/HCV rapid test (~ 1.5 USD) and an additional incentive (~ 1 USD) for each successful peer referral. We collected behavioral data via face-to-face interviews using a standardized behavioral questionnaire that included information on socio-demographic and social network characteristics, sexual and drug use behaviors, and substance use treatment. Participants also provided blood samples for HIV testing, with pre-test and post-test counseling. HIV testing was performed using SD-Bioline, South Korea rapid test, and if reactive, was followed by a confirmatory Unigold HIV rapid test.

### Study variables

The primary outcomes of this study were awareness and willingness to use HIVST. We provided a short description of an HIVST for the study participants: “The HIV self-test is an innovative and convenient method for HIV testing, which involves individuals performing the test on themselves using a small blood sample or oral saliva. It eliminates the need for laboratory equipment and can be conducted at home or any preferred location. However, in cases where a positive test result is obtained, it is crucial to seek confirmation through additional testing at a counseling center or medical facility for appropriate guidance for further steps”. We then measured the awareness and willingness to use HIVST by the following questions “Have you ever heard of an HIVST?” with the following response options (yes vs. no) and “Would you use an HIVST if it were made available in Iran?”, with the following response options: (a) very low willingness to use HIVST (b) low willingness to use HIVST, (c) high willingness to use HIVST, or (e) very high willingness to use HIVST. A binary variable of willingness to use HIVST was subsequently generated: (a) very low or low willingness to use HIVST, (b) high or very high willingness to use HIVST.

We examined correlates of willingness to use HIVST including socio-demographic, structural, and HIV and injection-related factors. Socio-demographics and structural covariates included: age (< 30, ≥ 30), gender (women, men), education (high school or more, less than high school), marital status (single, currently married, divorced/widowed), employment status (having a permanent job, having a temporary job, unemployed), lifetime history of homelessness (no or yes), lifetime history of incarceration (no or yes), experience of stigma within healthcare settings (never or ever), and experience of stigma from family/friends (never or ever). HIV- and injection-related variables included HIV risk perception (very low/low or moderate/high), duration of injection (< 5 years, 5–10 years, or > 10 years), injection frequency in the last three months (monthly or less, weekly or daily), receptive needle/syringe sharing in the last three months (yes or no), public injection in the last 12 months (yes or no), and using needle exchange programs in the last 12 months (yes or no).

### Statistical analysis

After excluding individuals who knew they were living with HIV, the analytic sample included study participants who responded to the question about willingness to use HIVST (n = 2,252). Descriptive statistics, including absolute and relative frequencies of the categorical variables, were used and reported to describe the characteristics of the study participants stratified by the willingness to utilize HIVST. Bivariable and multivariable modified Poisson regression models were used to examine correlates of willingness to utilize HIVST [23]. The selection of individual and environmental covariates in the bivariate and multivariable analyses was guided by the HIV risk environment framework [24], along with a review of relevant literature. To estimate and report the adjusted prevalence ratios (aPR) along with 95% CI, variables with a *P* value < 0.2 in the bivariable model were included in a full multivariable regression model [25]. A final model was selected through a backward elimination approach with significance set at *P* value < 0.05. These analyses were conducted in Stata v.17 (StataCorp, College Station, Texas, USA). Given that unweighted regression models offer greater accuracy, broader coverage, and more robust estimates compared to RDS-weighted models [26], we adopted an unweighted regression modeling strategy, consistent with a growing body of research [27, 28]. Additionally, we calculated and reported RDS-adjusted estimates for willingness to use HIVST by covariates, taking into account network size and homophily within networks. The RDS-adjusted estimates were computed using RDS-Analyst version 1.8.

### Ethical considerations

All study protocols were reviewed and approved by the Kerman University of Medical Sciences ethics committee (Ethics Codes: IR.KMU.REC.1397.573). Participation was anonymous. All participants provided verbal informed consent for biological and behavioral data collection before to study enrollment.

## Results

### Participant’s characteristics

Of the 2,252 participants in the analytic sample, the mean age was 40.1 (standard deviation [SD] = 9.3) years, and the majority of participants were ≥ 30 years old (89.0%; n = 1,992) and men (96.5%; n = 2,174). Over two-thirds (70.1%; n = 1,572) had less than high school education, 25.4% (n = 554) were married, and 18.0% (n = 342) had a permanent job. One-third reported moderate or high HIV risk perception (35.3%; n = 630). A large proportion reported lifetime experiences of homelessness (57.0%; n = 1,275), incarceration (66.9%; n = 1,469), stigma within healthcare settings (45.0%; n = 980), and stigma from family/friends (84.1%; n = 1,888). Moreover, over half (59.7%; n = 1,333) injected weekly or daily in the last three months, 4.1% (n = 83) reported receptive needle/syringe sharing in the previous three months, more than two-thirds (70.7%; n = 1,433) injected in public spaces in the last 12 months, and 88.0% (n = 1,522) received free needle/syringes in the last 12 months (Table 1). Additional file 1: Table S1 also presents RDS-adjusted estimates.

**Table 1:** Willingness to use HIV self-testing by socio-demographic characteristics, HIV- and injection-related factors, and harm reduction utilization among people who inject drugs in Iran (2020)

Variable	Total N (%)^a^	Willingness to use HIV self-testing			
		Very low or low n (%)	High or very high n (%)	Prevalence ratio (95% CI)^b^	*P* value
Overall	2,252	594 (26.4)	1,658 (73.6)	–	–
Age at interview, mean years (SD)	40.1 (9.3)	39.9 (9.5)	40.2 (9.2)	–	–
Age, years					
< 30	246 (11.0)	67 (27.2)	179 (72.8)	1	0.893
≥ 30	1,992 (89.0)	527 (26.5)	1,465 (73.5)	1.01 (0.86, 1.18)	
Gender					
Women	78 (3.5)	29 (37.2)	49 (62.8)	1	0.258
Men	2,174 (96.5)	565 (26.0)	1,609 (74.0)	1.17 (0.89, 1.57)	
Education					
Less than high school	1,572 (70.1)	443 (28.2)	1,129 (71.8)	1	0.141
High school or more	671 (29.9)	150 (22.3)	521 (77.7)	1.08 (0.97, 1.19)	
Marital Status					
Single	784 (35.9)	196 (25.0)	588 (75.0)	1	
Currently married	554 (25.4)	203 (36.6)	351 (63.4)	0.84 (0.74, 0.96)	0.012
Divorced/widowed	846 (38.7)	181 (21.4)	665 (78.6)	1.04 (0.95, 1.17)	0.407
Current employment					
Unemployed	45 (2.4)	20 (44.4)	25 (55.6)	1	
Having a temporary job	1,513 (79.6)	387 (25.6)	1, 126 (74.4)	1.33 (0.90, 1.99)	0.184
Having a permanent job	342 (18.0)	91 (26.6)	251 (73.4)	1.32 (0.87, 1.99)	0.148
HIV risk perception					
Very low or low	1,154 (64.7)	338 (29.3)	816 (70.7)	1	
Moderate or high	630 (35.3)	47 (7.5)	583 (92.5)	1.31 (1.18, 1.45)	< 0.001
Lifetime homelessness					
No	963 (43.0)	317 (32.9)	646 (67.1)	1	
Yes	1,275 (57.0)	275 (21.6)	1,000 (78.4)	1.17 (1.06, 1.29)	0.002
Lifetime incarceration					
No	740 (33.1)	257 (34.7)	483 (65.3)	1	
Yes	1,496 (66.9)	332 (22.2)	1,164 (77.8)	1.19 (1.07, 1.32)	0.001
Experience of stigma within healthcare settings					
Never	1,197 (55.0)	318 (26.6)	879 (73.4)	1	
Ever	980 (45.0)	265 (27.0)	715 (73.0)	0.99 (0.90, 1.10)	0.898
Experience of stigma from family/friends					
Never	357 (15.9)	127 (35.6)	230 (64.4)	1	
Ever	1,888 (84.1)	466 (24.7)	1,422 (75.3)	1.16 (1.02, 1.34)	0.028
Duration of injection, year					
< 5	545 (25.1)	224 (41.1)	321 (58.9)	1	
5–10	555 (25.5)	161 (29.0)	394 (71.0)	1.20 (1.04, 1.40)	0.013
> 10	1,073 (49.4)	187 (17.4)	886 (82.6)	1.40 (1.23, 1.59)	< 0.001
Injection frequency, last 3 months					
Monthly or less	901 (40.3)	299 (33.2)	602 (66.8)	1	
Weekly or daily	1,333 (59.7)	282 (21.2)	1,051 (78.8)	1.18 (1.07, 1.30)	0.001
Receptive needle/syringe sharing, last 3 months					
No	1,935 (95.9)	525 (27.1)	1,410 (72.9)	1	
Yes	83 (4.1)	20 (24.1)	63 (75.9)	1.04 (0.81, 1.34)	0.751
Public injecting, last 12 months					
No	593 (29.3)	212 (35.7)	381 (64.3)	1	
Yes	1,433 (70.7)	351 (24.5)	1,082 (75.5)	1.17 (1.04, 1.32)	0.007
Utilizing needle exchange program, last 12 months					
No	208 (12.0)	78 (37.5)	130 (62.5)	1	
Yes	1,522 (88.0)	373 (24.5)	1,149 (75.5)	1.20 (1.01, 1.44)	0.041

^a^N for each variable may not add up to total sample (2,252) due to missing value

^b^Prevalence ratios (PR), 95% confidence intervals (CIs), and *P* value were obtained from bivariable modified Poisson regression

### Awareness and willingness to use HIV self-testing

Overall, 362 (16.2%; 95% CI 14.7, 17.8) had ever heard of HIVST, however; 1,658 (73.6%; 95% CI 71.7, 75.4) reported high/very high willingness to use HIVST.

### Factor associated with willingness to use HIV self-testing

Willingness to use HIVST was significantly higher among PWID who had a high/moderate HIV risk perception compared to low HIV risk perception (92.5% vs. 70.7%; *P* < 0.001), ever experienced homelessness (78.4% vs. 67.1% for never; *P* = 0.002), had ever been incarcerated (77.8% vs. 65.3% for never; *P* = 0.001), and experienced stigma from family/friends (75.3% vs. 64.4% for never; *P* = 0.028). A significantly higher level of willingness to use HIVST was also reported among PWID were injected for more than ten years (82.6% vs. 58.9% for < 5 years; *P* < 0.001), injected weekly or daily in the last three months (78.8% vs. 66.8% for monthly or less; *P* = 0.001), reported public injecting in the last 12 months (75.5% vs. 64.3%; *P* = 0.007), and used needle exchange program services (75.5% vs. 62.5%; *P* = 0.041) (Table 1).

Multivariable analysis showed that willingness to use HIVST was higher among PWID with high/moderate HIV risk perception compared to low HIV risk perception (aPR 1.22; 95% CI 1.09, 1.37), those who ever experienced homelessness compared to never homeless (aPR 1.15; 95% CI 1.03, 1.28), those with > 10 years of injecting compared to < 5 years (aPR 1.16; 95% CI 1.00, 1.34), and those with weekly or daily injection frequency in the last three months (aPR 1.18; 95% CI 1.05, 1.32) (Table 2).

**Table 2:** Factor associated with willingness to use HIV self-testing among people who inject drugs recruited in 2020 national HIV bio-behavioral surveillance survey in Iran

Variables	Adjusted prevalence ratio^a^	95% CI	*P* value
HIV risk perception			
Very low or low	1		
Moderate or high	1.22	1.09, 1.37	< 0.001
Lifetime homelessness			
No	1		
Yes	1.15	1.03, 1.28	0.014
Length of injecting career			
< 5 years	1		
5–10 years	1.09	0.92, 1.29	0.293
> 10 years	1.16	1.00, 1.34	0.043
Injection frequency, last 3 months			
Monthly or less	1		
Weekly or daily	1.18	1.05, 1.32	0.005

^a^Using multivariable modified Poisson regression, variables with a *P* value < 0.2 in the bivariable analysis were entered into the multivariable analysis. The final model was selected through a backward elimination approach with significance was set at *P* value < 0.05

## Discussion

Despite low prior awareness of HIVST, the vast majority of PWID in Iran were willing to use HIVST to assess their HIV status. Willingness to use HIVST was significantly higher among PWID who reported a history of homelessness, high-frequency injection practices, a longer injecting history, and high/moderate HIV risk perception. The high willingness to use HIVST among PWID in Iran is consistent with findings reported among populations at risk for HIV in other international settings. For example, about half of transgender women in Malaysia reported that they would be willing to use HIVST [29]. A study among men who have sex with men in Mexico reported that 94% of participants would use HIVST if it were available [30]. Among people who use drugs (PWUD) in the US, 77% of PWUD in Louisville reported high acceptability of using HIVST [31], and 63% of PWUD in rural Appalachia were willing to take an at-home HIV test [32]. These data suggest a significant potential for the adoption of HIVST among PWID in Iran to increase HIV testing uptake and case identification, if implemented effectively.

Our analysis suggests that, when implementing HIVST, it is crucial to prioritize PWID with risky injection practices and those who have experienced homelessness due to their high-risk profiles and greater willingness to use HIVST. This increased willingness to use HIVST among high-risk and marginalized PWID supports the idea that HIVST can effectively reach hidden subgroups and at risk individuals who otherwise are unlikely to test for HIV [12]. Given the socioeconomic status of PWID in Iran (i.e., under one-fifth had a permanent job in our sample), programs should provide access to low-cost, affordable HIVST to increase testing frequency among this population in Iran [33]. Moreover, a higher willingness to use HIVST among PWID with a higher HIV risk perception, while most PWID reported a very low or low HIV risk perception, also suggests the need for programs to improve HIV risk perception among PWID in Iran. Having a low HIV risk perception can decrease the likelihood of HIV testing, and research has demonstrated that individuals with a low HIV risk perception may benefit from tailored interventions and provider-initiated strategies aimed at increasing their HIV perception of risk [34]. Implementing and increasing access to and awareness of HIVST can also serve as a way to improve HIV risk perception and increase HIVST uptake. Overall, the findings suggest the potential for effective integration of HIVST into Iran’s HIV prevention and treatment programs.

Research on both the barriers and recommendations for implementing HIVST in Iran is limited. However, several challenges and recommendations for the effective implementation and adoption of HIVST have been identified internationally. Studies have recognized cost as a barrier, affecting the impact of HIVST, willingness to test, and frequency of testing [35, 36]. This issue is particularly salient in low- to middle-income countries and among marginalized populations, such as PWID. For instance, a study among women receiving antenatal care and their male partners in Uganda revealed that testing costs profoundly influenced their preferences. Free testing was favored over tests priced at 2.00 USD or 2.90 USD [37]. Another study among truck drivers showed that even those who can afford an HIVST kit might be hesitant to purchase one [38]. Indeed, the longstanding practice of offering free HIV testing services, as offered in Iran [15], might have established a standard that could be challenging to change, and introducing charges for HIVST kits could hinder the effectiveness of this novel testing approach. It is suggested that HIVST should be made available free of charge or at a very low-cost, especially for marginalized individuals with lower incomes [39, 40]. The adoption of HIVST among high-risk subgroups may be impacted by the ease of accessibility, in addition to the costs [36]. A recent review of low- and middle-income countries highlighted that HIVST is a convenient alternative to facility-based testing, offering benefits, such as improved confidentiality, reduced stigma, and increased autonomy. However, the acceptability of HIVST generally depends on its affordability and accessibility to the target population [41]. Another review also indicated that adoption of HIVST is impacted by an individual’s perceptions of the costs, benefits, personal needs, and degree of convenience in accessing it, suggesting that oral HIVST is a better strategy as it is relatively painless when compared to HIV testing using finger-stick whole blood [36]. Furthermore, adapting HIVST to diverse populations, including PWID involves ensuring that programs, especially the instructional materials, align with the unique characteristics of the population, such as the level of literacy [42]. Studies also suggest that barriers to accessing counseling and ensuring successful linkage to care are essential issues that need to be addressed in the implementation of HIVST. To increase HIVST uptake and enhance counseling and linkage to care, partner- or peer-based distribution strategies are recommended [41, 43].

## Limitations

Our findings should be interpreted with a number of limitations. First, data were collected using face-to-face interviews, which may be subject to social desirability bias and underreporting of higher risk behaviors. Second, despite our efforts to recruit PWID from various geographical regions, our sample may not fully represent PWID across the entire country. Third, it should be noted that most PWID in our study had no prior experience or knowledge of HIVST, and thus, may not have been fully aware of the concept. This could have led to an underestimate of the prevalence of willingness to use HIVST. However, we did provide a brief explanation of HIVST to the participants before conducting the interviews. Lastly, data collected were cross sectional and causality cannot be inferred.

## Conclusion

Our study suggests that a substantial number of PWID in Iran are willing to use HIVST if it is available. PWID who were experiencing homelessness, involved in high-risk injection practices, and had a higher HIV risk perception were more willing to use HIVST, suggesting that PWID with high-risk profiles would be more willing to use HIVST. Given the low level of HIV testing among HIV key populations in Iran, HIVST provides a promising approach to help reduce stigma, decentralize services, increase testing, and reach the most hidden groups. The low awareness of HIVST highlights the need to enhance awareness through increased access to HIVST and health education programs. Additionally, conducting implementation science studies to effectively design and implement HIVST programs for PWID in Iran are warranted to help increase their access to HIV testing.

### Supplementary Information

**Additional file 1.** RDS-adjusted estimates for willingness to use HIV self-testing among people who inject drugs in Iran in 2020.

## Data Availability

Data will be available upon request submitted to the corresponding author (hsharifi@kmu.ac.ir).
