# Supplementary material for: Awareness and willingness to use HIV self-testing among people who inject drugs in Iran
Source: Harm Reduct J. 2023 Oct 7;20:145. doi: 10.1186/s12954-023-00881-z (PMC10560425; doi:10.1186/s12954-023-00881-z)
Supplement: Supplementary file 1 — Additional file 1. RDS-adjusted estimates for willingness to use HIV self-testing among people who inject drugs in Iran in 2020. [file 12954_2023_881_MOESM1_ESM.docx]

**Supplementary table 1.** RDS-adjusted estimates for willingness to use HIV self-testing by sociodemographic characteristics, HIV and injection-related factors, and harm reduction utilization among people who inject drugs in Iran in 2020.

| **Variable** |  |  | **Willingness to use HIV self-testing** | |
| --- | --- | --- | --- | --- |
|  | **Total N (RDS adjusted %)** | **Very low or low n (RDS adjusted %)** | | **High or very high n (RDS adjusted %)** |
| **Overall** | 2,252 | 594 (29.6) | | 1,658 (70.4) |
| **Age at interview, mean years [SD]** | 40.1 (9.3) | 39.9 (9.5) | | 40.2 (9.2) |
| **Age**, years |  |  | |  |
| < 30 | 246 (12.0) | 67 (26.5) | | 179 (73.5) |
| ≥ 30 | 1,992 (88.0) | 527 (24.1) | | 1,465 (75.9) |
| **Gender** |  |  | |  |
| Women | 78 (3.9) | 29 (39.7) | | 49 (60.3) |
| Men | 2,174 (96.1) | 565 (25.6) | | 1,609 (74.4) |
| **Education** |  |  | |  |
| Less than high school | 1,572 (73.3) | 443 (26.4) | | 1,129 (73.6) |
| High school or more | 671 (26.7) | 150 (20.3) | | 521 (79.7) |
| **Marital Status** |  |  | |  |
| Single | 784 (36.2) | 196 (26.6) | | 588 (73.4) |
| Currently married | 554 (25.9) | 203 (34.7) | | 351 (65.3) |
| Divorced/widowed | 846 (37.9) | 181 (29.9) | | 665 (70.1) |
| **Current employment** |  |  | |  |
| Unemployed | 45 (5.0) | 20 (40.0) | | 25 (60.0) |
| Having a temporary job | 1,513 (80.0) | 387 (22.3) | | 1, 126 (77.7) |
| Having a permanent job | 342 (15.0) | 91 (25.1) | | 251 (74.9) |
| **HIV risk perception** |  |  | |  |
| Very low or low | 1,154 (69.3) | 338 (30.0) | | 816 (70.0) |
| Moderate or high | 630 (30.7) | 47 (8.0) | | 583 (92.0) |
| **Lifetime homelessness** |  |  | |  |
| No | 963 (46.0) | 317 (36.8) | | 646 (63.2) |
| Yes | 1,275 (54.0) | 275 (20.9) | | 1,000 (79.1) |
| **Lifetime incarceration** |  |  | |  |
| No | 740 (37.1) | 257 (39.9) | | 483 (60.1) |
| Yes | 1,496 (63.9) | 332 (29.0) | | 1,164 (71.0) |
| **Experience of stigma within healthcare settings** |  |  | |  |
| Never | 1,197 (50.1) | 318 (30.0) | | 879 (70.0) |
| Ever | 980 (49.9) | 265 (29.0) | | 715 (71.0) |
| **Experience of stigma from family/friends** |  |  | |  |
| Never | 357 (16.1) | 127 (39.2) | | 230 (60.8) |
| Ever | 1,888 (83.9) | 466 (24.0) | | 1,422 (76.0) |
| **Duration of injection**, **year** |  |  | |  |
| < 5 | 545 (29.0) | 224 (45.5) | | 321 (54.5) |
| 5-10 | 555 (21.5) | 161 (30.0) | | 394 (70.0) |
| > 10 | 1,073 (49.5) | 187 (17.6) | | 886 (82.4) |
| **Injection frequency, last 3 months** |  |  | |  |
| Monthly or less | 901 (44.4) | 299 (37.8) | | 602 (62.2) |
| Weekly or daily | 1,333 (55.6) | 282 (25.2) | | 1,051 (74.8) |
| **Receptive needle/syringe sharing, last 3 months** |  |  | |  |
| No | 1,935 (96.9) | 525 (25.0) | | 1,410 (75.0) |
| Yes | 83 (3.1) | 20 (23.1) | | 63 (76.9) |
| **Public injecting, last 12 months** |  |  | |  |
| No | 593 (31.3) | 212 (39.9) | | 381 (60.1) |
| Yes | 1,433 (68.7) | 351 (24.0) | | 1,082 (76.0) |
| **Utilizing needle exchange program, last 12 months** |  |  | |  |
| No | 208 (12.8) | 78 (37.4) | | 130 (62.6) |
| Yes | 1,522 (87.2) | 373 (29.3) | | 1,149 (70.7) |
